# The Optimized Conformation Dynamics of the KcsA Filter as a Probe for Lateral Membrane Effects: A First Principle Based Femto-Sec Resolution MD Study

**DOI:** 10.3390/membranes12121183

**Published:** 2022-11-24

**Authors:** Johann Summhammer, Georg Sulyok, Gustav Bernroider, Massimo Cocchi

**Affiliations:** 1 Institue of Atomic and Subatomic Physics, Technische Universität Wien, Stadionallee 2, 1020 Vienna, Austria; 2 Department of Biosciences, University of Salzburg, 5020 Salzburg, Austria; 3 Department of Veterinary Medical Sciences, Università di Bologna, 40064 Bologna, Italy

**Keywords:** KcsA filter, conduction rates, conformational dynamics, lateral membrane effects, optimized channel geometry

## Abstract

We provide a high resolution, all-atom, femto-second molecular dynamics (MD) simulation of the passage of K^+^ ions and H_2_O molecules through the selectivity filter of the KcsA potassium ion channel, based on first principle physical methods. Our results show that a change in the length of the selectivity filter of as little as 3%, regardless of whether the filter is made longer or shorter, will reduce the K^+^ ion current by around 50%. In addition, further squeezing or stretching by about 9% can effectively stop the current. Our results demonstrate optimized conformational dynamics that associate an increased mobility of parts in the filter linings with a standard configuration, leading to maximized conduction rates that are highly sensitive to geometrical distortions. We discuss this latter aspect in relation to lateral membrane effects on the filter region of ion channels and the ‘force from lipids’ hypothesis.

## 1. Introduction

Functional aspects behind biological organizations strongly build on the electrostatic interaction between their constituent molecules. One prototypical example is provided by the structural and dynamical aspects of cell membranes, where the interplay between lipids and inserted membrane proteins can account for the regulation of many vital processes, including electrical signaling in nerve cells. A long sequence of studies has demonstrated that changes in the local composition of cell membranes can strongly modulate the ion-conductive properties of inserted membrane proteins, including voltage-gated channels by ‘lipo-electric interactions’. See [1] for a recent review. However, there are some natural restrictions in the experimental study of molecular interactions in the membrane. First, manipulations usually have to focus on isolated target molecules and this approach may not maintain the integrated membrane function that can be expected from their naturally highly disordered and dynamic environment in the living membrane. A helpful route to overcome this restriction has been made available by the addition of theoretical and computational tools to simulate the environment of highly resolved structures, starting with ‘(low-temperature) snapshots’ of single molecules and studying molecular dynamics (MD) through modifications of their interactions within a highly mobile environment, e.g., [2,3].

Initially, this approach was somewhat hindered by limited computational power. It showed that the characteristic time scale in molecular dynamics in general, under equilibrated conditions, is very short, i.e., in the range of fs (10^−15^ s), whereas experimentally observed events such as transmembrane currents are established in the microsecond to millisecond range (10^−6^–10^−3^ s). The possibility of bridging this large gap between very rare events and the typical time scale behind macroscopic ensemble averages of events has become attainable only more recently, through increased and parallel computational resources that can span the time differences without loss of decisive resolution.

The present report demonstrates the implementation of such simulations and the application to the study of lateral membrane effects exerted by the lipid-environment on voltage-gated ion channels. Among a wide range of possibilities that alter the lateral segregation of cell membrane molecules with possible consequences on the function of inserted proteins, the special role of poly-unsaturated fatty acids (PUFAs) has received particular attention [1,4,5]. PUFAs are found to directly regulate membrane channel function, with far reaching physiological and neuro-psychological consequences [6], although the precise mechanism is still somehow elusive [7]. The basic distinction that is frequently made in such a situation is to distinguish between two types of effects, i.e., ‘direct’ or ‘non-specific’ effects [1]. Here, ‘direct effects’ are defined as lipo-electric interactions between charged groups on the lipid (e.g., the negative charge on lipid head group carboxyls) on one hand, and the functional parts of the channel protein (e.g., the voltage sensor domain VSD of K^+^ channels) on the other [7,8]. ‘Non-specific membrane effects’ may operate very generally through the ‘force from lipids’ (ffl) principle [4], changing the conformation of channels through positive and negative pressure profiles. However, this distinction may eventually resolve as these effects can be narrowed down to the highest resolution of the target principle, the ionic-coordination and translocation through the atomic surrounding of the channel molecule.

**Figure 1:**
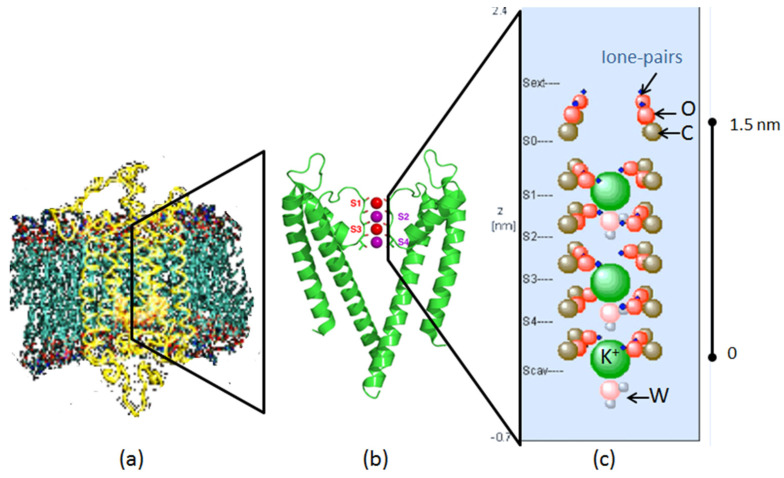
(**a**) The KcsA channel embedded in the cell membrane and surrounded by the lipid bilayer (adapted from [13] and modified from [14]). Protein as yellow ribbons, lipid tails as chains, water as sticks and K^+^ and Cl-ions as red and grey balls, respectively. (**b**) A stripped view of the KcsA channel with the mechanical gate at the bottom and the selectivity filter occupied by K^+^ ions (red and magenta) in the upper part. Only two strands of the tetrameric protein structure are shown. (**c**) The selectivity filter occupied with a sequence of K^+^ ions (green) and H_2_O molecules (pink and grey) in the K1WK3W configuration, with an additional K^+^ ion and a water molecule entering the filter from the cavity (S_cav_).

We focus on examining the functionally decisive domain of K^+^ channel proteins, the selectivity filter (SF), which has received great attention ever since its crystallographic characterization around the year 1998 [9]. The SF is formed by a tetramer of subunits with a highly conserved sequence of amino acids and carbonyl groups oriented towards the narrow filter pore (Figure 1). This configuration allows for four transient binding sites for filter ions (‘oxygen-cages’) that are largely responsible for the domain’s preference for certain ions (e.g., K^+^ over others e.g., Na^+^). Selective and conductive properties are assumed to be optimized by the delicate geometries between interacting charges along the filter atoms. How this arrangement can engage in selective binding while maintaining high conduction rates has been disputed by a sequence of papers in the last few years, e.g., [10,11]. However, a ‘very high resolution’ view on filter dynamics reveals a new aspect that has not previously been detected by employing potentials from mean force field (PMF) methods and time scales in the nano to micro-second range. If ion translocation is simulated from first-principle methods at atomistic resolution with the channel held in the open-gate state as in the present approach, ion transfer appears to be clearly confined to single ‘bursts’ with a characteristic time in the pico-second range. This picture meets both properties of the channel as it renders the filter states ‘non-conductive’, but potentially selective over extended time intervals in the range of ns, but also allows conduction rates to add up to about ~10 ions/ns and to the experimental diffusion limit of ~10^8^ ions/s [12]. Much of our focus in this article will be on the role of the emerging optimized geometry underlying this conduction and the possible environmental effects that can distort the associated dynamics. In other words, we examine the extent to which the present atomistic MD model of the KcsA SF can respond to perturbations arising from its surrounding molecular forces exerted by lipid compositions.

First, we provide a detailed description of the SF based on the prototypical KcsA motive and the implementation of the present MD approach with forces derived from first-principle methods. We then discuss the emerging dynamics with special attention to an optimized structure and its sensitivity to linear distortions along its longitudinal transmembrane direction, as this distance is seen to shorten or extend, depending on the gating state of the channel from atomic force microscopy [15]. Finally, we discuss the results with relation to the ‘force from lipids’ and ‘lipo-electric’ concepts, particularly when applied to the role of PUFA interactions.

## 2. Materials and Methods

### 2.1. The Selectivity Filter of the KcsA Potassium Channel

The selectivity filter of the KcsA K^+^-channel is formed by a five-residue sequence, the amino acids Threonine (Thr75), Valine (Val76), Glycine (Gly77), Tyrosine (Tyr78), and Glycine (Gly79), commonly abbreviated as TVGYG, which is highly conserved from bacteria to human cells (Figure 1). It is located within the P loop of each of the four subunits of the tetrameric structure of the ion channel proteins. Under physiological K^+^ concentrations, the selectivity filter residues arrange to form rings of carbonyl oxygen atoms directed roughly towards the center of the pore axis. Four adjoining sites can be defined and are usually designated as S1–S4. Dehydrated K^+^ ions bind in these cage-like sites. Further binding sites for partially or fully hydrated K^+^ ions have been also identified at the extracellular entrance (S0, S_ext_) and towards the central cavity (S_cav_) of the channel [16,17].

### 2.2. Modeling the Selectivity Filter

Our model of the KcsA selectivity filter (SF) is shown on the right-hand side of Figure 1. The SF was a nano pore of about 1.5 nm in length, which was made up of four strands of peptide chains. On each chain we found six essential carbonyl groups. We took the carbon atoms of these carbonyls (shown as brown spheres) to be at fixed positions. Each oxygen atom of a carbonyl (oxygens are shown as red spheres) had a constant distance to its carbon, but it was able to vibrate around its mean position in both angular directions. The carbon atoms had a positive partial charge and the oxygen atoms had a negative partial charge of equal magnitude (for details see the Appendix A). The charge of a carbon atom was centered, but the charge on the oxygen was dispersed over two points, in order to represent the lone pairs electrons of the oxygen, which play an important role in holding and transporting the K^+^ ions and the water molecules which must diffuse through the SF. Therefore, one part of the charge of the oxygen was centered on the atom itself, but the lone-pairs part was a point charge slightly outside of the atom (shown as blue dots in Figure 1, right image). It is always located along the line of the C-O-axis, even when the O-atom swings around the C-atom.

The mobile species in the SF were the K^+^ ions (green spheres) and the water molecules (pink are oxygen atoms and attached grey hydrogen atoms). The K^+^ ion carried one unit of positive charge. They were only able to move along the central axis of the SF. In the water molecules, the oxygen atoms had a negative partial charge and the hydrogen atoms each had a positive partial charge of half the magnitude, so that the water molecule was neutral but had an electric dipole moment. The water molecules, too, were only able to move along the central axis of the SF. A water molecule can have two principal orientations: either the oxygen atom is on top, or a hydrogen atom is on top. In addition, a water molecule can have an arbitrary rotational angle around the central axis of the SF. Further details of the model, especially the forces acting between the various particles, are presented in the appendix.

### 2.3. Motion of K^+^ and H_2_O through the SF

**Figure 2:**
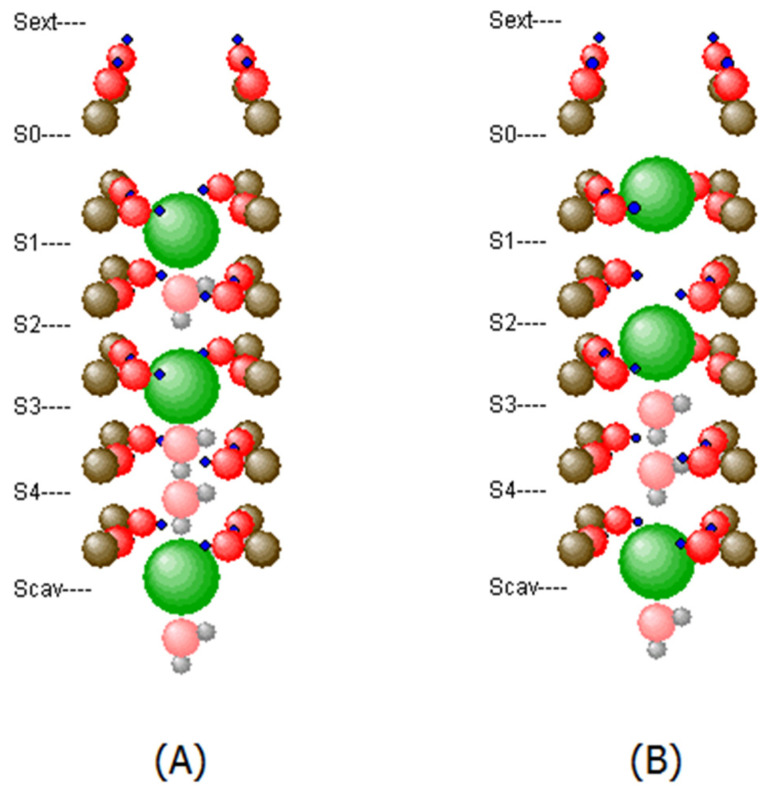
(**A**) Example of a filter state (K1WK3-WWKcav) which is characterized by extended stability with no ion emission over periods around 100 ns; (**B**) In contrast the occupancy pattern (K0K2WWKcav) is highly unstable and conductive, with inter-emission intervals around 1–2 ns.

The calculation of the motion of K^+^ and H_2_O, as well as the vibrating motions of the oxygen atoms was carried out using methods of molecular dynamics based on the Verlet algorithm. The software was developed by us, in order to allow the incorporation of a quantum mechanical motion of particles, as reported in [18] previously. The calculations focus on the situation when the gate at the inner side of the membrane is open (Figure 1, middle picture) and ions and water can enter the channel from the inside of the cell to the outside. Therefore, there was a sufficient supply of K^+^ ions and water molecules on the lower side of the SF. The K^+^ in the cavity before the SF are usually in a hydrated state. For a K^+^ to enter the SF, it must get rid of its shell of water molecules. During this process, it is also possible for H_2_O molecules to enter the SF. Therefore, there is a pressure from K^+^ as well as from H_2_O to enter the SF. These partial pressures are assumed to be equal and hence there is an equal chance for either a K^+^ or an H_2_O to hop into site S_cav_ as soon as this is sterically possible. The partial pressure from the K^+^ ions is at the origin of the electric potential difference between the inside and the outside of the channel, because on the outside this pressure is kept at a lower value. The motion of both species through the SF is mainly governed by the behavior of the K^+^ ions, for which the SF is a series of potential barriers and valleys constituted by the electric charges. The K^+^ have certain sites S_cav_, S4, …, S0, where the lone-pair electrons of the oxygens of eight carbonyl groups coordinate around the ion, in very similar way to how water molecules would coordinate around a K^+^ immersed in water. The K^+^ can be attached to such a site for a relatively long time. However, in the model, the whole structure of the SF was assumed to be in the thermal bath of the surrounding proteins, and therefore the O-atoms of the carbonyl groups exhibited random motion in addition to the motions which they underwent due to their interaction with the K^+^, the water molecules, but also with other O-atoms and C-atoms. The temperature of this bath was kept at 310 K. Therefore, this thermal motion was also conveyed to the K^+^ and to the H_2_O, so that these particles were able to overcome the barrier to the next site and hop into it coincidentally. If at such a moment, all sites of the SF are occupied, this is only possible in a concerted action. If a site is free, then only a single K^+^ or H_2_O from a neighboring site may move and hop into the site. In this case, both directions of motion are possible, because the pure electric field due to the difference in K^+^ concentration between inside and outside of the channel is too small to inhibit backwards motion. The motion of the K^+^ ions through the SF is therefore a stop-and-go motion. In a typical KcsA channel in the open condition, the ion current is approximately several 10^8^ ions per second. On average, only every 2 to 5 nanoseconds a K^+^ ion leaves the SF on the upper side, and since occupancy at any given time is between 2 and 4 K^+^ ions, an average ion spends between 6 and 15 nanoseconds in the SF. On the other hand, it takes only around 1 picosecond to hop the distance of around 0.3 nm from one site to the next. Theoretically, the SF could thus be traversed within 5 to 10 picoseconds, but in fact it takes about 1000 times longer. This is due to the strong coordination forces of carbonyl oxygens and their lone-pair electrons, which hold up a K^+^ at every site within the SF. Figure 2A,B show two occupancy patterns of the SF which had an exceptionally long and a rather short hold-up time of the K^+^ current, respectively.

Water molecules as such could possibly traverse the SF much faster on their own, because their dipole forces do not pull them strongly to the carbonyls. However, since they are mostly trapped between two K^+^ ions and passage through the SF is only possible in single files, their flow rate is essentially the same as that of the K^+^ ions.

### 2.4. Lateral Membrane Effects

**Figure 3:**
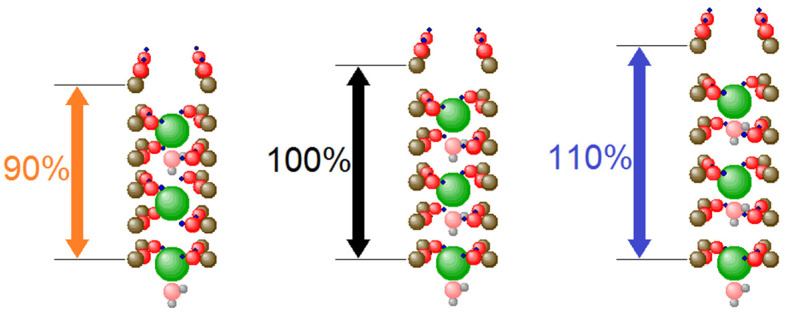
Changing the length of the selectivity filter. Simulations were made for different lengths, ranging from 92% to 110% of the ‘standard length’.

Here we want to look at a possible influence on the selectivity filter of such channels, which is a much smaller structure. Just as in the larger structure of the ion channel as a whole, two effects can be envisaged. The changed concentration of PUFAs can either lead to the build-up of additional charges near the SF, or the changed local pressures can distort the geometry of the SF, and it is also conceivable that both effects act simultaneously. In the present paper, we want to concentrate on the influence on geometry, and in particular we want to look at the stretching and squeezing of the SF along its central axis. In contrast to a radial squeezing or stretching, which would either lead to a quench of the ion current or to a strong increase when the nano pore becomes wider, it is not immediately clear what can be expected if the sites S0 to S4, at which K^+^ ions are bound for longer periods of time, are pulled farther apart or pressed closer together. Therefore, we made simulations in which the relative distances of the carbon atoms parallel to the central axis of the SF are modified. Figure 3 gives a graphic presentation of the changes made.

**Figure 4:**
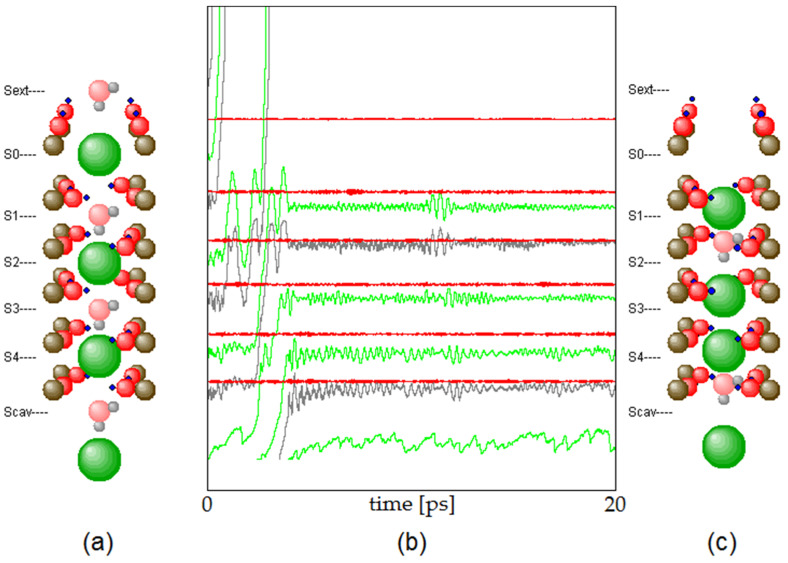
Initial configuration (**a**) and configuration after thermalization within 20 ps (**c**). The graph (**b**) shows the z-component (upwards) of the momentary positions of K^+^ ions (green), H_2_O molecules (grey) and O-atoms (red). Note that two K^+^ and two H_2_O exit the SF on top within the first few ps. Within about the same time, two K^+^ enter below from the cavity region and an H_2_O and a K^+^ enter the site S_cav_ from the cavity, ready to enter the SF as soon as the particles at higher position move upwards. The vibrational amplitude of the O-atoms is relatively small due to the stiffness and corresponding high frequency of the C=O bond (wavenumber around 500 cm^−1^).

At the beginning of a simulation of the current flow through the SF, the carbon atoms, together with the associated oxygen atoms, were set to positions adapted to the chosen filter length. K^+^ ions and water molecules were placed into the SF, as shown in Figure 4a. In order to achieve a thermalized configuration, a thermalization period of 4 ns was started based on a Verlet time step of 0.25 fs, so that a dynamical evolution over 4 million time steps could take place. During this period, a sufficient number of ions and water molecules usually passed through the SF to reach a thermal equilibrium of all the degrees of freedom of the involved particles. Thermal equilibrium was frequently reached within a much shorter time, as can be seen in Figure 4c. After thermalization, the actual simulation of interest was launched, which covered a period of 10 µs, again using a Verlet time step of 0.25 fs. The times of injection into and emission from the SF of K^+^ ions during this evolution were recorded for statistical evaluation. For each of the investigated SF lengths, eight such simulations were run.

## 3. Results

The present simulations provide insight into the question of whether the stretching or squeezing of the selectivity filter can have an effect on K^+^ ion conductance, given the atomic geometry of a standard open-gate KcsA-SF model. Stretching or ‘squeezing’ are implemented as symmetrical variations of linear distances between backbone atoms along the longitudinal, i.e., axial direction, of filter atoms. Lateral forces influencing this geometry can be expected to arise mainly from the outer leaflet atoms of the surrounding lipid bilayer [1] (see discussion).

The fs-resolution MD model implemented on an atomic scale, as reported here, can detect the smallest changes in the vibrational modes of the interacting atoms. These modes come with changes in the electronic distribution of neighboring atoms, e.g., with the bending and stretching of carbonyl oxygen lone pairs, as previously reported by density functional studies [19] and Brownian dynamics simulations [20]. The interaction term between ions and electrons within the filter is therefore basically ‘*vibronic*’, i.e., ionic motions change the electronic states of the molecule during their transitions. Details of this interaction will be discussed elsewhere. In this study (e.g., Figure 4), we can observe the oscillatory behavior of ions and intermittent water molecules in the range of THz (10^12^ Hz). These oscillations ‘couple’ with almost zero-lag time correlations to their immediate neighbors in the filter under unperturbed conditions (e.g., during long periods without changes in the cavity location of ions, and ion exits from the filter). Such synchronizations become particularly stable in the K1,3 configuration (e.g., K1W2K3WW), where K denotes the K^+^ ion, the number denoting the binding site location, and W represents water molecules, (see Figure 2). Relatively unstable configurations leading to ion exits from the channel mostly involve K2 and K4 occupancy states, which is in accordance with early studies suggesting a ‘blocking-gate’ in the SF with the K1,3 occupancy and all transits involving the K2,4 configuration [21].

### 3.1. Optimized Conduction Rates at Standard Configuration

**Figure 5:**
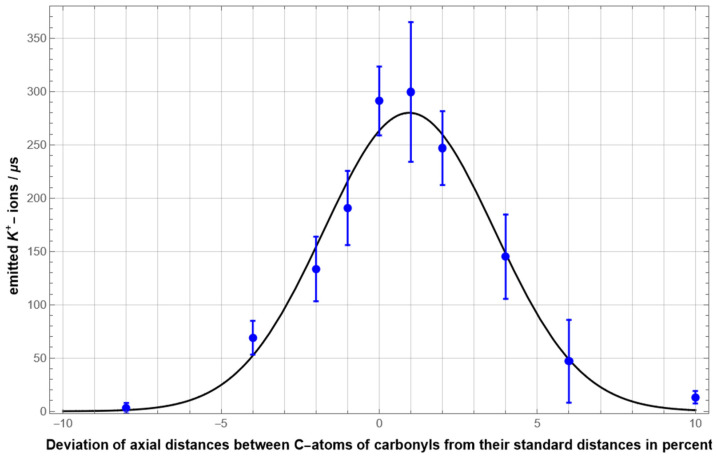
The K^+^ ion current as a function of length of the selectivity filter. Each blue dot marks the mean value obtained from 8 molecular dynamics simulations of the selectivity filter, each enduring 10 µs. The error bars indicate the related standard deviations. The black curve demonstrates a Gaussian fit to these data (Fit function: $I\left(x\right)=I_{0}e^{-{\left(x-x_{0}\right)}^{2}/2w^{2}}$, with fit parameters $I_{0}=280.0$ ions/µs, $x_{0}=0.949$ percent, w=2.70 percent).

Figure 5 summarizes the results of simulations involving modifications of the longitudinal extension of the SF ranging from 92% to 110% of the standard length. For each extension, eight simulations of 10 µs duration with a time resolution of 0.25fs were carried out. The highly notable result is that the K^+^ ion current became strongest when very close to the ‘standard distance’, and that it dropped off dramatically for longitudinal length changes in both directions. When the SF was extended or compressed by only about 3% from the “maximal ion current length” the ion current was already reduced by one half. When stretching or compressing the selectivity filter further, ion flow practically stopped completely, at +/− 9% length deviation.

For ions in an aqueous solution flowing through a (cylindrical) nanopore, resistance depended linearly on the pore length. Thus, one would usually expect an increased ion flow when the pore length is reduced. Our result is counterintuitive at first sight and requires detailed investigation and explanation on the microscopic level, which will be given in the upcoming sections. It is worth noting that although the reduction of the ion current for an increased filter length may be expected qualitatively from the simple pore model, the high degree of current reduction was remarkable and only explicable from the microscopic interaction between ions and filter atoms as well.

### 3.2. Coordination Mobility

**Figure 6:**
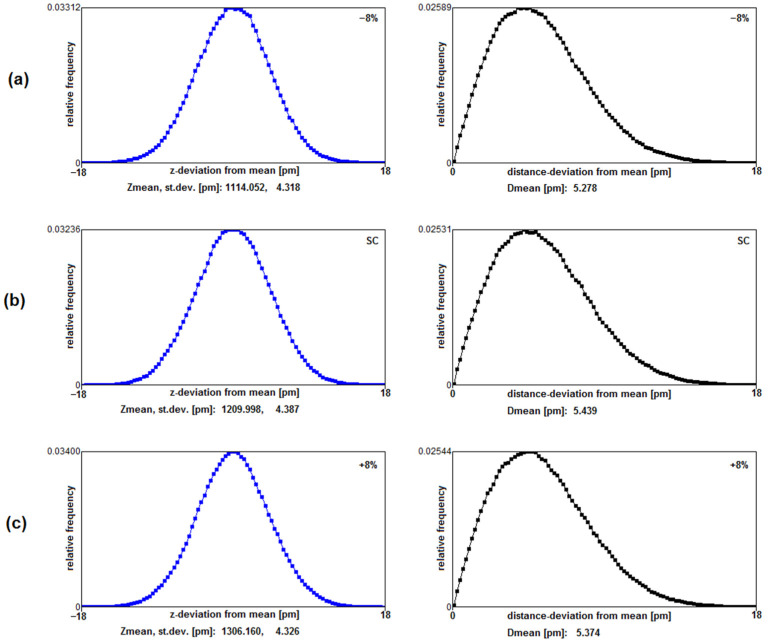
(**a**–**c**) Frequency distributions for z-deviations from mean oxygen locations and total distance deviations from mean oxygen locations of the Tyr78 carbonyl oxygen (the lower boundary of location S0) for a K0W1K2W3K4 configuration. Results for standard distances of the filter are given in (**b**), and those for 8% shorter or longer filter in (**a**,**c**), respectively.

It is not immediately conceivable why the standard geometry behind the studied Coulombic interaction distances should provide a maximum conduction rate. We have not found clear differences in the average kinetic barrier heights or total potential energy of ions between the different axial extensions of the filter. A subsequent investigation of parts of the lining carbonyl mobility, however, indicates a possible mechanism that favors high throughput under standard coordination distances at locations S1 to S0 of the filter. Starting with a high occupancy state of 3 K^+^ ions in the filter at locations K0W1K2W3K4 and W (as seen from outside to inside), we calculated the deviation from mean locations of the Tyr78 derived carbonyl oxygen atoms during 600 ps of evolution. (In the present simulations these deviations are caused by fluctuations of the C=O bond angle and not by the bond length). The comparison was made between the standard configuration and length changes of ±8% filter length and in each case involved 2.4 × 10^6^ x,y,z O-coordinates. Figure 6, below, provides a comparison of relative frequencies for deviations from mean z- coordinates and from the mean of total distances along all spatial orientations. Measures are given in pm. 

**Figure 7:**
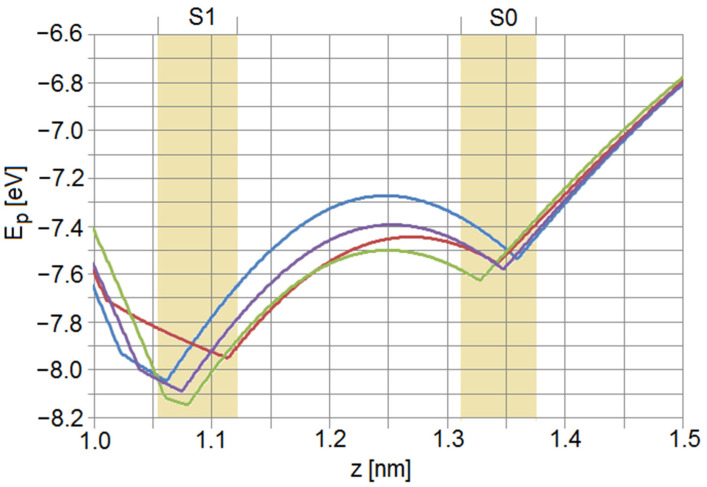
Four snap shots of potential Ep of a K^+^ ion in the region between S1 and S0 taken within a few ps. The filter has occupancy K0W1K2W3K4 and standard length as in the central image of Figure 3. The fluctuations of the carbonyl oxygens, which are essentially Boltzmann-distributed by random exchanges with the thermal bath, lead to constantly changing barrier heights between S1 and S0. In these examples they range between 0.51 eV and 0.77 eV (19–29 k_B_T), in agreement with other simulations in, e.g., [11,22].

It is apparent from the above carbonyl fluctuation study that at least one critical property given by the deviations of oxygen distances from their resting location seems to favor the exit of ions from their outermost filter location. The distance deviation from the mean along all coordinates was 5.439 pm for standard length and decreased to 5.278 pm for −8% (Figure 6a) and 5.374 pm for +8% filter length changes, respectively. A closer look at the distributions in the right column reveals that, e.g., an oxygen deviation of the mean of more than 12 pm occurred for about 2.14% of the time for standard filter length, while it was only 1.87% for the shorter filter and 1.97% for the longer filter. While these fluctuations might seem small, one has to consider that the point charge, which in the simulations represents the lone-pair electrons (blue dots at the oxygen atoms in Figure 2, Figure 3 and Figure 4) extends about 82 pm deeper towards the axis of the filter than the center of the oxygen atom, and has correspondingly larger spatial fluctuations. The joint motion of the carbonyl oxygens and their respective lone pairs can then lead to quite large fluctuations in the effective barrier height between the sites S1 and S0, as shown in Figure 7. Therefore, it is expected that an increased mobility of coordinating oxygens may provide an increase in conduction-favoring moments at locations close to the filter exit, and this situation is favored by a ‘standard’ Coulombic geometry guiding ions through the filter. 

## 4. Discussion

We demonstrated the structure and basic dynamical principles of the KcsA SF using an atomistic MD model employing a series of simulations, each of which extended over 4.10^10^ time steps of 0.25 fs and involved all fixed and mobile atoms with direct ion-interactions. In the course of manipulations of the geometrical properties underlying the control of conduction through filter atoms, we found an *optimized arrangement*. ‘Optimized’ in the present (mathematical) sense should be interpreted involving the interplay between three variables, the maximization of some function (a) provided by variables (b) in the context of a set of constraining principles (c). The function (a) is given by the conduction rate of the SF, the variables (b) are presented by the alignment of interaction distances in the SF and the constraining principles involve the stability, selectivity and energetic principles of the SF. The results summarized in Figure 5 clearly identify the linear longitudinal extension along the alpha-helical backbone of four strands with five residues, each lining the filter region, as the most sensitive variable controlling conduction. The ‘standard’ measure of this length maximizes conduction while maintaining stability and selectivity. Even a very small change in this length along both directions in the range of a few percent will significantly reduce conduction rates, and changes around 10% of this length eventually stop conduction completely.

The high sensitivity of the filter length to changes in axial extension may render the atomic configuration particularly susceptible to perturbations from surrounding side chains, gating and lipid composition. The KcsA channel is known to shorten its longitudinal length upon opening the pore-gate of the protein. In addition to the crystallographic picture, the study of Sumino et al. confirms these changes by atomic force microscopy (AFM) of the naturally in-situ membrane-embedded channel [15]. These changes amount to about 20% with the ‘short’ version representing the open-gate structure. To what extent these modifications upon gating will affect the geometry of the SF and the associated force field remains to be seen.

The observed symmetrical Gaussian type of conduction rate changes with filter length modifications (Figure 5) along both axial directions, together with the observation that length changes do not reflect changes in total ionic potential energies, are particularly in need of an explanation. What we find in the present study is an increased mobility of carbonyl oxygens in standard configurations compared to filter length deviations. The following consideration may shed some light on why this occurs: While for a shorter-than-normal filter length carbonyl-oxygen vibrations may be damped by the closer proximity to a K^+^ ion, a longer-than-normal filter length may decouple the oxygen vibrations from the relatively large vibration amplitudes of the K^+^ ion. The location of these conspicuously mobile carbonyls is close to the filter exit and lined by two glycine derived carbonyl sites. This observation could be seen in the context of previous solid-state NMR studies that associate an enhanced backbone mobility of these two glycines in a membrane-embedded environment with the conduction property of a KcsA filter motif [23]. These results, originating from studies using spectroscopic methods, support the present finding that an increase in the mobility of carbonyls, particularly at upper part locations of the filter, close to the exit of ions, may in fact play a causal role in changing the conduction property of the filter. 

Within this context, a clear difference between atomic scale dynamics and methods based on averages of conduction becomes important. Computational methods based on mean field averages such as Poisson—Nernst—Plank (PNP) and Brownian Dynamics (BD) studies are insensitive to the high-resolution dynamics induced by surface charges in very narrow ionic channels. With the increase of computational power, however, high resolution MD simulations can provide a deeper insight into single atom vibrational and electronic interactions. Earlier studies incorporating the motion of single charges from lining carbonyl atoms into Brownian dynamics simulations have already given an indication that carbonyl fluctuations can have an effect on the slope of conduction in the filter [20]. This increase in conduction becomes apparent from rms fluctuations starting with 0.1 pm (0.01 Å), which is within the range of mean distance deviations as reported here. These observations become even more important in the pico-second view of single conductive events. As reported before [14], conduction occurs largely within single ‘bursts’ at the pico-scale. Once an expulsion of an ion occurs it is likely that others follow. For these events only short time-windows are sufficient, i.e., in the range of pico-seconds, hosting about 20–50 oscillation periods of carbonyls at a wave-number of k = 500 [cm^−1^] as employed here (see the Table A1 in Appendix B for a demonstration of frequencies, oscillation periods, bending constants and associated energies for different wave numbers of carbonyl fluctuations). With the above observation (Figure 6) that at standard filter length large amplitude oscillations are about 10% more frequent compared to longer or shorter filter extensions, it can be assumed that this type of fluctuation at location S0 of the filter can at least favor the observed maximum conduction rate under normal geometrical conditions.

The present simulation study of an all atom-lining Coulombic interaction geometry indicates optimized conformation dynamics in the highly preserved filter composition of voltage-gated K^+^ channels. We might, in addition, expect that the present level of simulation studies might still resolve even more accurately through the combination of classical and quantum descriptions of ionic transition, as suggested by the authors in a number of contributions before [24,25].

Finally, we want to make a note on the physiological significance behind the tight cooperation of an ‘active membrane’ regulating the interplay between lipids and ion channels as seen from the present work. The far-reaching physiological effects of PUFAs, together with their dietary role, have received great attention during the few last years. In the context of neural function, some PUFAs such as Linoleic Acid (LA) and alpha Linolenic acid (ALA) have been found to be of relevance for psychiatric conditions such as depression, as discussed with relation to evolutionary aspects by one of the co-authors, M. Cocchi [26]. However, a clearly defined site of action and the precise mechanism within the lateral heterogeneity of the membrane still have to be enlightened. In the traditional ‘Fluid-Mosaic Model’ of Singer and Nicholson [27], which has dominated the perspectives for almost half a century, these aspects are not well accommodated [5]. The noticeably low concentration of LA and ALA around 1–10 µM in membranes has invoked different interpretations about a specific mechanism controlling channel function, particularly within the superfamily of K^+^ channels. Cocchi et al. provide a ‘symmetry breaking’ model in which low linoleic concentrations might operate at a boundary between ‘phase-transitions’ that could eventually affect larger membrane domains across cells and spread into the modular level of the entire brain [28]. 

A connection between the level of conductive sensitivity of K^+^ channels as seen from the present results and the phase-transition hypothesis exerted by PUFAs mentioned above could possibly pave a path to the urgent question of channel cooperativity in the brain. However, this insight is difficult to obtain because of the inhomogeneous composition of membranes and a lipid polymorphism that strongly depends on a large set of environmental, physical and chemical parameters, reviewed in [29]. Generally, tension and pressure are governed by the balanced interaction of two opposing forces, a contracting force within the lipid head groups and an expanding force in the membrane interior between the lipid acyl tails. It is generally assumed that this interplay results in a typical transmembrane lateral pressure profile with a negative pressure at membrane interfaces and a smaller positive pressure profile in the interior [18]. It has been shown that this pressure profile can also differentially affect the conductive properties of membrane inserted KcsA potassium channels that serve as a model for the present study [30]. In most previous studies, however, it is generally assumed that proteins respond with functional changes to lateral pressures if these mechanistic forces can induce a conformational change of the protein that results in a depth-dependent variation of cross-(horizontal) sectional areas of the protein. Our present MD simulation, however, demonstrates that the central selectivity domain with the highly conserved lining structure among voltage-gated K^+^ channels can change conduction rates without essential conformational changes and no in-depth variation of sectional areas. This suggests a new view of the possibility of mechanistic membrane forces modulating selective conduction. It suggests a *dynamic criticality* of ion channel proteins provided by an optimized *‘readiness’* for conduction without the loss of selectivity. Mechanic forces that change this critical extension will have a cooperative effect on ionic diffusion and the emerging electrical membrane signals. However, it must be conceded at this stage that we can only present indirect evidence from high resolution MD simulations, and further evidence involving direct spectroscopies would help to elucidate this conjecture further.

## Figures and Tables

**Figure A1:**
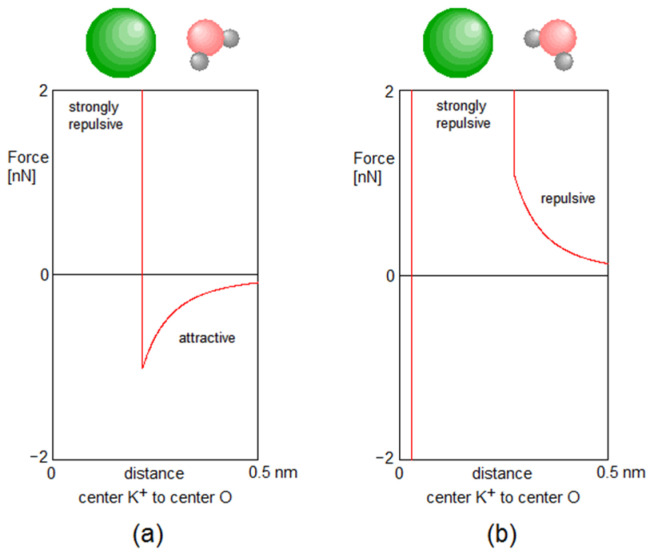
Force between a K^+^ ion and a water molecule as a function of distance. (**a**) The negative oxygen atom of the water molecule points towards the positive K^+^ ion. As long as the distance between the center of K^+^ and of the water molecule is larger than 0.248 nm (about 90% of the sum of the radius of K^+^ and the effective radius of H_2_O), the total coulomb force between the K^+^ and the three charges of the water molecule is attractive with a maximum magnitude of almost 10^−9^ N. Within the repulsion zone an extremely strong repulsive force sets in, however. This configuration occurs when the water molecule is part of a hydration shell of the K^+^ ion. When this configuration occurs within the selectivity filter, which is often the case when a water molecule from the inner cell cavity follows a K^+^ ion into the selectivity filter, the water molecule becomes attached to the K^+^ and moves with it through the selectivity filter. (**b**) One positive hydrogen atom of the water molecule points towards the positive K^+^ ion. Here the total Coulomb force between the K^+^ and the water molecule is always repulsive, but at a distance of 0.5 nm it has fallen off to only about 2 × 10^−10^ N. However, if the water molecule and the K^+^ approach closer than the repulsion zone and the electron shells begin to overlap, the very strong repulsion force sets in.

**Figure A2:**
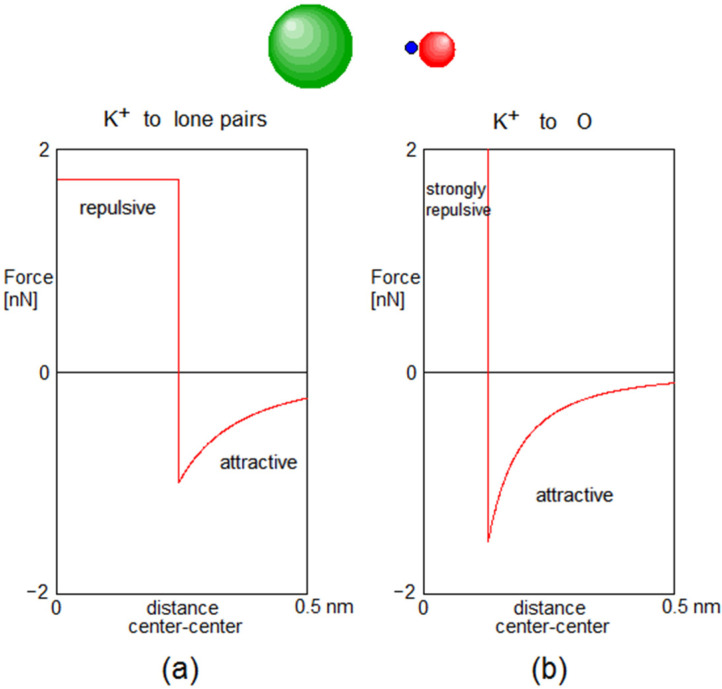
Force between a K^+^ ion and the oxygen atom of a carbonyl group. As we modeled the oxygen atom as two-point charges, where one also carries mass (the actual O-atom), and the other no mass but only a charge (the center point of the lone pairs charge distribution) one must consider separately the force between the lone-pair electrons of the oxygen atom and the K^+^ (picture (a)) and the force between the actual oxygen atom and the K^+^ (picture (b)). (**a**): When the positive K^+^ approaches the negative electron lone-pairs point charge, there is a mutual attractive force due to the Coulomb interaction, which can reach almost 10^−9^ N. At a center-center distance of the two charges of 0.238 nm, however, the strong but constant repulsion of orbital overlap sets in, with a magnitude of about 1.7 × 10^−9^ N, which corresponds to an energetic cost of full overlap of K^+^ and the electron lone-pairs charge density cloud of 100 k_B_T (T = 310 K). (**b**): When the positive K^+^ approaches the negatively charged main body of the oxygen atom, the Coulomb force is attractive and reaches a value of about 1.5 × 10^−9^ N. At center-center distances of K^+^ and O of less than 0.124 nm the orbitals (other than the lone-pair electrons of the oxygen) begin to overlap and a very strong and constant repulsion of 1.72 × 10^−8^ N sets in, which corresponds to an energetic cost of 500 k_B_T for full overlap of K^+^ and O. During the simulations, however, this repulsion never occurs, because the K^+^ are constrained to the central axis of the selectivity filter and never get close enough to the oxygen atoms.

**Figure A3:**
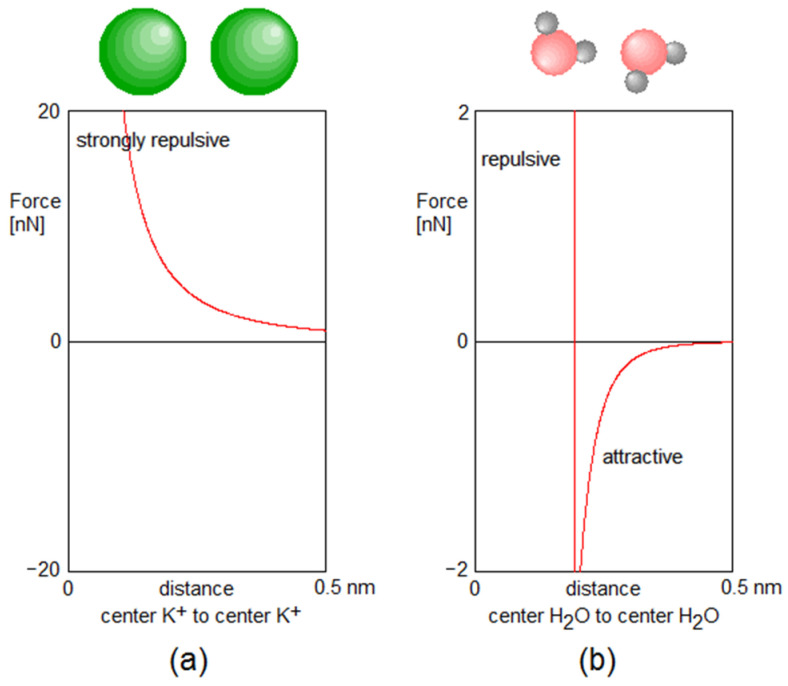
(**a**) Force between two K^+^ ions as a function of distance. This is a pure repulsive Coulomb force. Since each K^+^ carries +1 q0, the repulsion is so strong that an energy of 5.22 eV, or almost 200 k_B_T at 310 K, would be required for the two charges to approach each other to a center-center distance of 0.276 nm, where theoretically additional repulsion due to overlap of electron shells should set in. Such a close approach practically never happens at this temperature, and therefore no additional repulsive force had to be taken into account. (**b**): The force between two water molecules depends on their relative orientation and on their distance. Here, we show the case where the negatively charged oxygen of one molecule points to one of the positively charged hydrogens of the other molecule. The resulting attractive force pulls the two molecules together until they enter the hydrogen bond. Any closer approach would cause a strong repulsive force.

## Data Availability

The data presented in this study are available on request from the corresponding author.

## Appendix A. Forces between Particles

The ‘particles’ in the model of the selectivity filter are carbon atoms (C), oxygen atoms (O), potassium ions (K^+^) and water molecules (H_2_O). All of these particles carry full or partial charges, or have an electric dipole moment. Therefore, there may be Coulomb attraction or repulsion between them. However, if any two of them get too close and their electron shells begin to overlap, there will always be repulsion. In the following we will describe the most important mutual forces between the particles.

**Carbon atoms:** The C-atoms are fixed at the backbone protein strands and are rigidly immobile during a simulation. However, as each C is a member of a carbonyl group (C-O), it carries a partial charge of +0.38 q_0_ (q_0_…electric charge unit), and therefore exerts a Coulomb force on other charged particles. It was not necessary to consider short-range repulsive forces between the C-atoms and other particles, because no particle could get near enough to a C-atom.

**Oxygen atoms:** Each O-atom is a member of a carbonyl group and is attached to its C-atom with the known center-to-center distance of 0.123 nm. When at rest, each O-atom points in a certain direction from its C-atom. This direction is adjustable by the two angles of spherical polar coordinates. For the simulations, these angles were set to the typical values found in the literature [25]. It should be mentioned that these angles are usually such that the C-O axis does not point straight to the axis of the selectivity filter, as one might expect if the O-atoms are to form cages for the K^+^ ions. This can be seen in Figure 1, Figure 2 and Figure 3, e.g., at the outermost positions the C-O axis points upwards, while for the inner C-O groups it is horizontal and points to either the left or the right of the axis of the selectivity filter. Each O-atom can perform rotational vibrations around its two rest angles. The frequency of these vibrations is assumed to be the same for the two angles, and it is also assumed to be the same for all carbonyl groups. It was set to 500 cm^−1^ in accordance with values reported before [17]. These rotational vibrations also have a damping factor, which is supposed to model the dissipation of energy from the selectivity filter to the protein backbone. Such dissipation is necessary because when a K^+^ ion hops from one site to the next, they acquire kinetic energy when settling into the new site. In general, however, they can be caught by the new site only, if this energy is dissipated.

The O-atoms also connect all mobile species within the selectivity filter to a thermal bath. This is achieved by random accelerations or decelerations on the rotational vibrations of the O-atoms, such that the mean energy contained in these motions corresponded to k_B_T/2 per degree of freedom. Here, k_B_ is Boltzmann’s constant and the temperature T was set to 310 K.

In order that each carbonyl group is electrically neutral, each O-atom carries a charge of −0.38 q_0_. This charge is modelled as two-point charges, of which one resides in the center of the O-atom and the other is somewhat outside the covalent radius of the atom, but on the line defined by the C-O axis. It represents the effective charge of the electron lone pairs of the O-atom. In the simulations, this charge had a distance of 1.4 covalent radii from the center of the O-atom and carried 70% of its partial charge. Thus, a charge of −0.266 q_0_ was in the lone-pairs point and −0.114 q_0_ in the center of the O-atom. The lone-pairs charge point was assumed to be fixed on the line of the C-O axis, so it followed the rotational vibrations of the O-atom.

**K^+^ ions:** Each K^+^ carried an electric charge of +1 q_0_. It interacted with other particles by the Coulomb force. However, if another particle got closer to a K^+^ than the sum of the covalent radius of that particle and the ion radius of K^+^ (set at 0.131 nm), a constant repulsive force set in. The strength of this force depended on the kind of the other particle, which could be an electron lone-pairs charge, an O-atom or a water molecule. Generally, it was scaled such that a complete overlap of the center points of the two particles would consume energy of several hundred to a thousand k_B_T. Therefore, no substantial overlap between a K^+^ and any other particle ever occurred during the simulations. The motion of the K^+^ ions was constrained along the central axis of the selectivity filter. (This constraint was necessary to keep computation time at a manageable level).

**H_2_O:** The water molecules are modelled as three-point charges arranged in the usual triangular fashion. The point charge corresponding to the oxygen atom carries a partial charge of −0.7 q_0_ and the two-point charges corresponding to the hydrogen atoms each carried a charge of +0.35 q_0_. The distance of the hydrogen charge points to the oxygen charge point was set at the known distance between the corresponding nuclei in the water molecule, 95.84 pm. The angle between the bonds of the hydrogens to the oxygen was set to 104.5°. In order that the water molecules would not penetrate any other particles, a water molecule was also endowed with a constant repulsive force, which set in whenever any other particle did get too close. The border surface within which this force started to act was assumed to be spherical, in good agreement with the shape of the outer electron shell of the water molecule, and the center of this sphere was on the symmetry axis of the water molecule along the way between the oxygen and the hydrogens. Just like for the K^+^ ions, the motion of the hydrogen molecules was constrained to the central axis of the selectivity filter. In order to save further computational time, the orientation of a hydrogen molecule was limited. One of the O-H axes was always aligned with the central axis of the selectivity filter, with either the hydrogen or the oxygen atom pointing upwards. The rotational orientation of the other hydrogen atom around this axis could be at any value between 0° and 360°, however.

### Examples of Forces between the Particles

In the following we give some graphical examples of the forces between the particles. The action of the forces is explained in the figure captions.

## Appendix B

**Table A1:** Carbonyl Oscillations at Different Wave Numbers.

k [cm^−1^]	f [Hz]	Oszillations-Period [fs]	Bending Constant [N/rad]	Energies for $\theta_{0}=0.0813\;rad$ (ca. 0.1 Å)	
				E [J]	E [meV]
300	9 × 10^12^	111.1	1.0443 × 10^−8^	4.2450 × 10^−21^	26.50
400	1.2 × 10^13^	83.3	1.8565 × 10^−8^	7.5467 × 10^−21^	47.11
500	1.5 × 10^13^	66.7	2.9007 × 10^−8^	1.1791 × 10^−20^	73.60
600	1.8 × 10^13^	55.6	4.1771 × 10^−8^	1.6980 × 10^−20^	105.99
700	2.1 × 10^13^	47.6	5.6855 × 10^−8^	2.3112 × 10^−20^	144.27
